# Population genetic evidence for sex‐specific dispersal in an inbred social spider

**DOI:** 10.1002/ece3.2200

**Published:** 2016-07-12

**Authors:** Deborah R. Smith, Yong‐Chao Su, Reut Berger‐Tal, Yael Lubin

**Affiliations:** ^1^ Department of Ecology & Evolutionary BiologyUniversity of KansasLawrenceKansas66045; ^2^ Biodiversity InstituteUniversity of KansasLawrenceKansas66045; ^3^ Blaustein Institutes for Desert ResearchBen‐Gurion University of the NegevSede Boqer CampusMidreshet Ben‐Gurion84990Israel

**Keywords:** dispersal, inbreeding, population structure, sociality, spider

## Abstract

Dispersal in most group‐living species ensures gene flow among groups, but in cooperative social spiders, juvenile dispersal is suppressed and colonies are highly inbred. It has been suggested that such inbred sociality is advantageous in the short term, but likely to lead to extinction or reduced speciation rates in the long run. In this situation, very low levels of dispersal and gene flow among colonies may have unusually important impacts on fitness and persistence of social spiders. We investigated sex‐specific differences in dispersal and gene flow among colonies, as reflected in the genetic structure within colonies and populations of the African social spider *Stegodyphus dumicola* Pocock, 1898 (Eresidae). We used DNA fingerprinting and mtDNA sequence data along with spatial mapping of colonies to compare male and female patterns of relatedness within and among colonies at three study sites. Samples were collected during and shortly after the mating season to detect sex‐specific dispersal. Distribution of mtDNA haplotypes was consistent with proliferation of social nests by budding and medium‐ to long‐distance dispersal by ballooning females. Analysis of molecular variance and spatial autocorrelation analyses of AFLPs showed high levels of genetic similarity within colonies, and STRUCTURE analyses revealed that the number of source populations contributing to colonies ranged from one to three. We also showed significant evidence of male dispersal among colonies at one site. These results support the hypothesis that in social spiders, genetic cohesion among populations is maintained by long‐distance dispersal of female colony founders. Genetic diversity within colonies is maintained by colony initiation by multiple dispersing females, and adult male dispersal over short distances. Male dispersal may be particularly important in maintaining gene flow among colonies in local populations.

## Introduction

Permanent group‐living (social) species run a risk of inbreeding, which could have detrimental fitness effects on offspring by increasing the likelihood of homozygosity of rare, lethal recessive alleles or more numerous weakly deleterious alleles (Charlesworth and Charlesworth 1987). Inbreeding could also have negative effects on fitness by decreasing the likelihood of high‐performing heterozygous genotypes (Charlesworth 2003), leaving inbred individuals less able to respond to parasites, pathogens, or other environmental challenges. Cooperatively breeding birds and mammals avoid consanguineous mating largely through sex‐biased dispersal (Greenwood 1980; Greenwood and Harvey 1982; Pusey 1987). Many social insect species have mating swarms or mating flights in which males and females from different colonies may meet and mate, for example, bumble bees (Wilson 1971, pp. 87–88; Michener 1974, p. 326); stingless bees (Wilson 1971, p. 92; Michener 1974, pp. 131–132, 344–346); honey bees, (Wilson 1971, pp. 96–98; Michener 1974, pp. 361–362); ants (Holldobler and Wilson 1990, pp. 145–147); and termites (Wilson 1971, pp. 113, 115). In addition, kin recognition and behavioral avoidance of mating among close relatives are known to occur in social species across the animal kingdom (Pusey and Wolf 1996). Nevertheless, regular inbreeding occurs in some group‐living organisms (e.g., naked mole rats, Reeve et al. 1990; seed beetles, Gottlieb et al. 2009), and it remains unclear to what extent gene flow maintains low levels of genetic variation in such species.

Cooperative social spiders (henceforth, social spiders) are remarkable for exhibiting extreme inbreeding within colonies and limited dispersal among colonies. This behavior has evolved independently as many as 18 times in six different families of spiders (Agnarsson 2006; Lubin and Bilde 2007; Bilde and Lubin 2011), yet it remains exceedingly rare, occurring in 0.05% of spider species. This suggests that the behavior is advantageous in the short term but likely to lead to extinction or reduced speciation rates in the long run (Avilés 1997; Agnarsson et al. 2006, 2013; Johannesen et al. 2007). Despite their disparate origins, social spider species share a suite of behavioral and life‐history characteristics (Lubin and Bilde 2007; Bilde and Lubin 2011). They form colonies containing a few to thousands of individuals of both sexes. Sex ratio is strongly female biased, which reduces effective population size. Colony members cooperate in prey capture and web‐building, and some species also share care of young.

Social spiders also share key features of their population structure. Dispersal of offspring is suppressed and the young mature within their natal colony. Population genetic studies of several social spider species have shown that mating occurs primarily among colony mates and that there is low genetic variation within colonies and across the species, compared with related solitary species (Lubin and Crozier 1985; Smith 1986, 1987; Roeloffs and Riechert 1988; Smith and Engel 1994; Smith and Hagen 1996; Johannesen et al. 2009, 2012; Smith et al. 2009; Settepani et al. 2014). New colonies are initiated by division or budding of existing colonies, or by one or a few adult mated females dispersing by walking or ballooning.

This presents an obvious puzzle. If colonies are initiated by one or a few mated females, adult dispersal is local and mating occurs strictly among colony mates, then genetic drift should result in colonies comprised of nearly identical colony mates. Each population should exhibit low genetic diversity, high levels of fixation, and strong differentiation from other isolated populations. Early allozyme studies of the social species *Anelosimus eximius* (Keyserling, 1884) (Theridiidae) produced results partly consistent with these predictions: very low frequency of polymorphic loci, a small number of fixed differences between populations east and west of the Andes, and most colonies composed of individuals identically homozygous at all 52 allozyme loci examined (Smith and Hagen 1996). Agnarsson et al. (2010) found that the majority of *A. eximius* colonies they sampled in Ecuador and French Guiana were comprised of individuals sharing a single mtDNA haplotype, indicating descent from a single maternal lineage. However, meta‐population approaches to the study of social spider population structure (Avilés 1997; Settepani et al. 2014), more extensive sampling of species and populations, and more detailed genetic data from newer genetic techniques showed that some genetic diversity is maintained within colonies (e.g., *Stegodyphys dumicola,* Smith et al. 2009) and that genetic continuity across populations can be maintained through colony extinction and replacement by occasional immigration of long‐distance dispersers (Settepani et al. 2014).

Thus, the amount of variation maintained across a species will depend on the frequency of colony extinction and initiation, and the mobility and dispersal distance of colony initiators (see e.g., Settepani et al. 2014). Frequent colony extinction and replacement by new colonies initiated by long‐distance dispersers can maintain genetic similarity among populations and at the same time lead to lower genetic diversity across the species relative to outbreeding species.

The level of genetic variation maintained within a single social spider colony will depend upon the amount of variation present among colony founders, the colony's effective population size, colony life span, or number of generations that the colony persists, and whether or not the colony receives migrants into the pool of reproductive individuals. In essence, genetic variation within a colony depends on initial genetic diversity and subsequent effects of genetic drift and migration.

Given this population structure, very low levels of dispersal among colonies may have unusually important impacts on the fitness and persistence of social spiders. Thus, understanding mechanisms and level of gene flow among colonies (via females or males) and among populations is essential to understand how these inbreeding social systems are maintained.

Occasional gene flow among colonies could occur by at least four means. First, small single‐female nests established by ballooning females are sometimes joined by 1–2 additional dispersing females (Lubin et al. 2009) that might be of different origins. Second, ballooning females might land near and join an established colony. Social spiders generally tolerate the introduction of individuals from other colonies (Christenson 1984; Darchen and Delage‐Darchen 1986; Seibt and Wickler 1988a,b). Third, colonies of different origins could meet and fuse. Daughter colonies produced by repeated colony growth and budding can result in an expanding cluster of colonies, some of which might meet and fuse with colonies from a neighboring lineage. Finally, males might travel to nearby colonies in search of females of a different source population.

However, detecting and measuring migration among colonies has proved difficult. Here, we used genetic tools to investigate sex‐specific differences in dispersal as reflected in the genetic structure within colonies and populations of *Stegodyphus dumicola* Pocock, 1898 (Eresidae), an African social species for which considerable information is already available on their social organization, dispersal patterns, and population genetics. We used DNA fingerprinting and mtDNA sequence data along with spatial mapping of colonies and compared male and female patterns of relatedness within and among colonies at three study sites. Using the fine‐scale genetic data, we demonstrate how gene flow among colonies of a social spider may be maintained by sex‐specific dispersal.

## Methods

### Natural history

*Stegodyphus dumicola* occurs in savanna habitats in drier parts of southern Africa. Colonies are patchily distributed throughout the landscape and often clustered with many nests in close proximity (Seibt and Wickler 1988b; Lubin and Crouch 2003; Smith et al. 2009). Individual colonies usually consist of a cohort of a few to hundreds of individuals, of which about 10–20% are males. Reproduction is seasonal, and while the life span of individual females is approximately 1 year, males survive only 4–6 weeks as adults (Seibt and Wickler 1988b). *Stegodyphus dumicola* juveniles mature and mate within the colony; dispersal of individual juveniles out of a colony has not been observed.

Colony proliferation may take place by budding or by dispersal of mated adult females. In budding, a group of juvenile females and males moves a short distance (usually less than 5 m) away from a large colony and establishes a new daughter colony (Seibt and Wickler 1988b; Birkhofer et al. 2014). Initially, the webs may remain joined by silk lines and spiders move freely between these nests (Lubin et al. 2009). Large nests may also split due to branch fall or other disturbances and form adjacent daughter colonies. Both result in clustering of closely related colonies (colony lineages) at a site.

Colony initiation may also take place by adult, mated females dispersing individually from large colonies by aerial silk threads (ballooning) and establishing an incipient colony where they land (Seibt and Wickler 1988b; Schneider et al. 2001; Birkhofer et al. 2014). The distance traveled is variable; some settle near their parent colony, while others may be carried over long distances to initiate populations in a new location. Local populations of nests – formed by budding and by local ballooning – last for several years, but eventually most nests in a local population will become extinct (Lubin and Crouch 2003).

Long‐distance male mating dispersal is unlikely, as adult males have not been observed to balloon. The female‐biased primary sex ratio is also consistent with low male mating dispersal (Lubin et al. 2009). However, male dispersal over short distances has been demonstrated experimentally between a central colony and daughter colonies up to 7 m distant (Lubin et al. 2009). Under natural conditions, males might move to nearby nests in search of mates, particularly when females in the male's home nest have already mated and are less receptive. In nearby nests, they are likely to encounter females of the same maternal source.

### Collections

**Figure 1:**
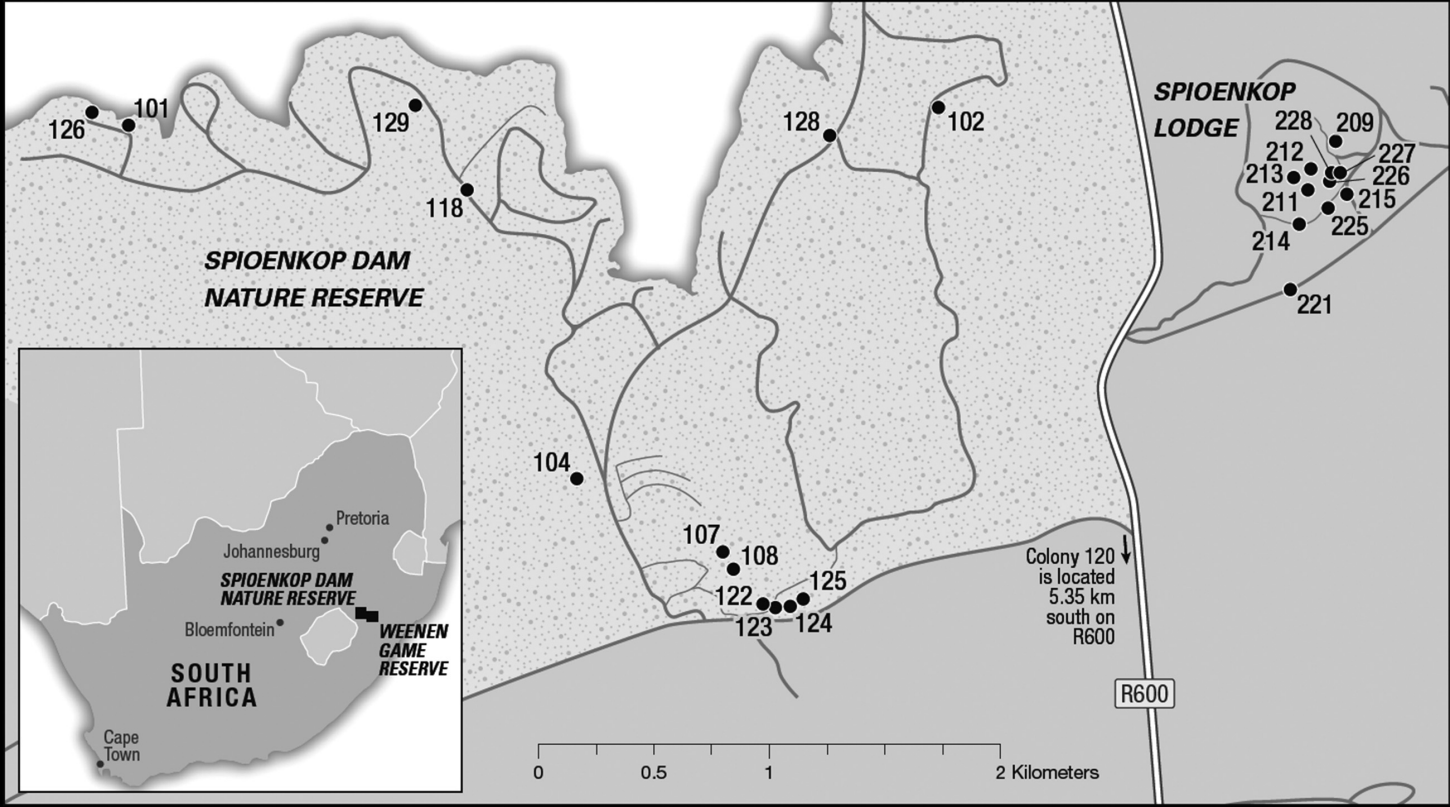
Map of *Stegodyphus dumicola* colonies at Spioenkop Dam Nature Reserve (100‐series samples) and Spioenkop Lodge (200‐series). Inset: location of Spioenkop and Weenen collection sites in South Africa.

**Figure 2:**
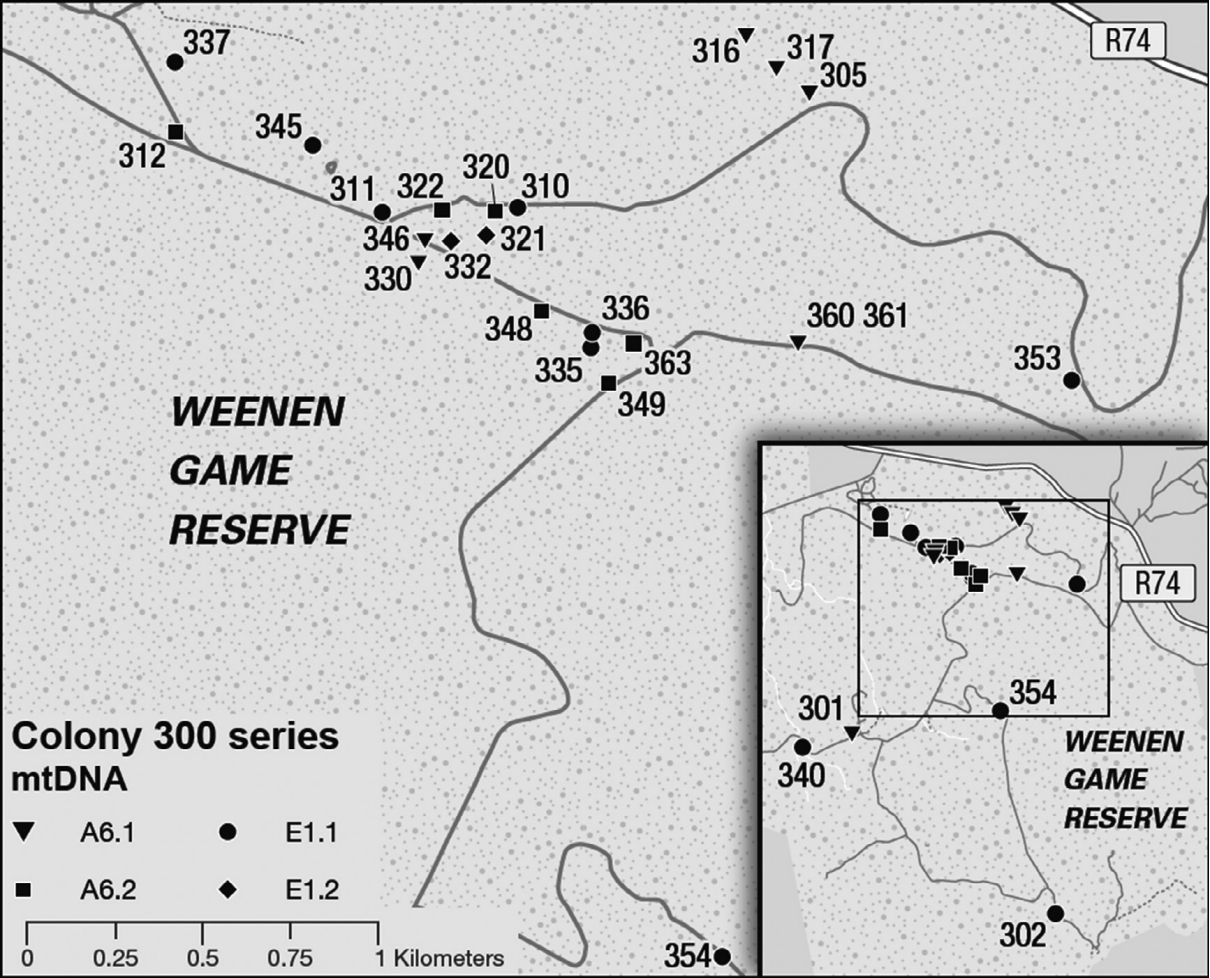
Map of *Stegodyphus dumicola* colonies (300‐series samples) at central Weenen collection site, showing mtDNA haplotype found in each colony. Inset: outlying colonies at Weenen site, showing mtDNA haplotypes found.

*Stegodyphus dumicola* were collected (permit #4546/2007 from Ezemvelo KZN Wildlife) from three sites in KwaZulu‐Natal Province, South Africa: Spioenkop Dam Nature Reserve, Spioenkop Lodge, and Weenen Game Reserve (Figs. 1 and 2). The habitat at all sites is wooded savanna. Samples (up to 11 females and up to 14 males per colony) were collected near the end of the mating season, when both males and females were present in most nests. Individual spiders were stored in 90–95% ethanol at or below −20°C.

### Mapping

GPS and hand mapping data for each colony were converted to *x*‐ and *y*‐coordinates in the UTM (Universal Transverse Meridian) grid system or on arbitrary maps. Matrices of pairwise geographic distances among colonies were constructed using GenAlEx 6.5 (Peakall and Smouse 2012).

### Genetic methods

#### DNA extraction

DNA was extracted from a leg or a portion of the cephalothorax of each individual using DNEasy (Qiagen, Valencia, CA) or GenElute Mammalian Genomic DNA Purification (Sigma‐Aldrich, St. Louis, MO) kits, following the manufacturers’ protocols. The remaining tissues were reserved as vouchers stored at the Blaustein Institutes for Desert Research (Ben‐Gurion University of the Negev, Sede Boqer Campus, Israel).

#### mtDNA sequencing

A portion of the mitochondrial genome comprising a portion of the ND1 and lsrRNA genes was amplified using primers N1‐J‐12261 (Hedin 1997) and LR‐N‐12945 (Simon et al. 1994) and sequenced on an Applied Biosystems 3130XL. Resulting sequences were aligned with published sequences from *S. dumicola* (Johannesen et al. 2002, 2009) in MEGA5 (Tamura et al. 2011).

We sequenced at least one female and up to 11 males from each colony, plus all individuals in small propagule webs (webs containing 1–3 dispersing individuals). For pairs of colonies with different mtDNA haplotypes and separated by 150 m or less, we sequenced up to four additional females from each colony.

#### mtDNA analyses

We tested whether neighboring colonies were more likely to be of the same haplotype or different haplotype using the 22 colonies in the “Central Weenen” area shown in Figure 2; we omitted four outlying colonies with no close neighbors. We ranked colonies by distance to nearest neighbor and noted whether the nearest neighbor was of the same or different haplotype. We then used information on nearest neighbor distances in the Weenen population to select distance classes for a spatial autocorrelation analysis (implemented in GenAlEx 6.501, Peakall and Smouse 2012), to determine whether colonies separated by short distances (50 m or less) were more likely to share the same mtDNA haplotype than pairs of colonies separated by greater distances. Pairwise genetic distances (1 or 0) indicated only whether colonies had the same or different mitochondrial haplotypes. Map data were also used to compare the haplotypes of individuals in small propagule nests to the nearest large nests.

#### DNA fingerprinting

Individual spiders were fingerprinted using the three‐enzyme‐amplified fragment length polymorphism method (TE‐AFLPs; see van der Wurff et al. 2000 for complete protocols and sequences of the adaptors and primers). Amplified DNA fragments were sized on an Applied Biosystems 3130XL, and resulting data were scored using Applied Biosystems GeneMapper 4.0 (Life Technologies, Grand Island, NY). Each amplified DNA fragment or “band” is represented by a discrete fluorescence peak.

#### AFLP band‐calling

Duplicate samples of several individuals were included in runs to check for consistency of band‐calling. To accurately recognize fluorescence peaks that correspond to amplified DNA fragments (to call bands), we followed Whitlock et al. (2008) to normalize peak heights across individuals and across loci, to select thresholds for accepting a peak, and to call phenotypes. We eliminated loci for which only one individual differed from the others sampled (“singletons”).

#### AFLP Analyses

We used the TE‐AFLP data to investigate overall population structure of our South African populations for comparison with earlier studies of Namibian populations (Johannesen et al. 2002; Smith et al. 2009) and to compare population structure and dispersal in males and females. Population structure was assessed using AMOVA (analysis of molecular variance; Excoffier et al. 1992) implemented in the program GenAlEx 6.5 (Peakall and Smouse 2012).

AMOVA generates Φ statistics, analogous to *F* statistics commonly used for codominant genetic data. One set of analyses addressed the partitioning of genetic variation among individuals, colonies, and collection sites; each collection site was considered a region, and within collection sites, each colony was considered a population. We analyzed (i) all colonies from which at least two individuals were sampled, males and females combined; (ii) females only, all colonies from which two or more females were sampled; and (iii) males only, all colonies from which two or more males were sampled. A second set of analyses explicitly compared partitioning of genetic variation among colonies for males *versus* females. Each collection site was analyzed separately, males and females were treated as two separate “regions,” and colonies were treated as all male or all female populations. The AMOVA statistic Φ_pt_ (analogous to *F*_ST_) measures the similarity of pairs of individuals from the same population (here, the same colony) relative to pairs of individuals drawn randomly from the total sample. Φ_pr_ measures the similarity of pairs of individuals drawn from the same population (colony), relative to individuals in the same “region” (here, either collection site or sex). Φ_rt_ measures the similarity of individuals drawn from the same “region” compared with pairs of individuals drawn randomly from the total sample.

Spatial autocorrelation analysis measures correlations between pairs of individuals (here, TE‐AFLP phenotypes) as a function of distance. We investigated whether males and females show significantly different patterns of autocorrelation, particularly within colonies. Spioenkop Lodge and Spioenkop Reserve collections were combined to avoid small sample sizes. We used the Spatial package in GenAlEx 6.501 (Peakall and Smouse 2012) with the Multiple Populations and Variable Distance Class options. We examined differences in the patterns of spatial autocorrelation of males and females using the methods of Smouse et al. (2008) and Banks and Peakall (2012) implemented in the Heterogeneity option. The statistics T2 and Omega (Banks and Peakall 2012) were used to evaluate departure from null expectations at individual pairwise distance classes (T2) and over the entire “correlogram” or plot of autocorrelation *r* values as a function of distance (Omega). The 95% confidence interval about the null hypothesis of no spatial structure for the combined data set was determined by 9999 permutations of the data. The 95% confidence interval about each *r* value was determined by 10,000 bootstrap resamplings. As recommended by Banks and Peakall (2012), we required *P* < 0.01 to consider a result significant.

We used a Bayesian model‐based clustering method implemented in STRUCTURE 2.3 (Pritchard et al. 2000) to test the underlying population genetic structures at the three collection sites. Colonies were used as the population subdivision prior for the informative location prior methods of Hubisz et al. (2009). We set the *a priori* ancestry numbers, *K*, from *K* = 1 to *K* = 10, and carried out 10 independent Markov chain Monte Carlo (MCMC) runs for each value of *K*. In each MCMC run, we discarded the first 50,000 states as burn‐in and collected the data from the second 50,000 states. Then, for each individual, we computed the proportion of AFLP markers originating from each inferred ancestor under each K. This could also be viewed as an individual's probability of being assigned to each ancestry. STRUCTURE outputs were submitted to the CLUMPAK cluster (Kopelman et al. 2015) to determine the optimal *K* using the second order likelihood change rate (or delta *K*) method as proposed by Evanno et al. (2005).

After determining the optimal *K* at each of the three collection sites, we used a fractional multinomial logit model (Papke and Wooldridge 1996) implemented in STATA 13 (module developed by Buis 2008) to test whether an individual's probability of being assigned to a particular ancestry differed among colonies or between sexes using the model “ancestry proportions = constant + sex + colony.” Here, an individual's sex and colony membership are independent variables, and the individual's probability of assignment to each of *K* ancestries is dependent variable. The fractional multinomial logit model is a quasi‐maximum‐likelihood method that models dependent variable(s) as proportions ranging from 0 to 1, with values for each individual summing to one. A significant statistical outcome would indicate that ancestries are different among colonies or between sexes. This analysis does not test for differences in ancestry between male and female colony mates.

## Results

### Mitochondrial data

Four mitochondrial haplotypes, here called A6.1, A6.2, E1.1, and E1.2, belonging to previously reported haplotype families A (in Namibia, Johannesen et al. 2002) and E (in South Africa, Johannesen et al. 2009), were found among our samples (GenBank KX363490, KX363491, KX363488, KX363489). All colonies at Spioenkop Reserve (14 colonies, 16 females, and 15 males) and Spioenkop Lodge (10 colonies, nine females, nine males) shared the same mtDNA haplotype, E1.1; all four mtDNA haplotypes were observed in the Weenen collection site (26 colonies, 67 females, 61 males). All males and females genotyped from the same colony shared the same mtDNA haplotype, even if the colony was located 150 m or less from one with a different mitochondrial haplotype.

We examined a total of 11 propagule nests from Weenen, each containing one or two females. Distance from propagule to nearest large nest ranged from 1.4 to 26 m. In most cases, individuals in propagule nests shared the mtDNA haplotype of the nearest large nest; however, in two cases, the spiders in the propagule nests carried a different mtDNA from the nearest large nest: propagule 311b, located 6.6 m from colony 311, and propagule 349b, collected 12.3 m from colony 349. One propagule nest from Spioenkop Reserve contained two females and one male, demonstrating natural male dispersal in this population.

Figure 2 shows the distribution of mtDNA haplotypes among colonies at the Weenen site. We used nearest neighbor distances among colonies to select distances of 50, 500, and 3000 m for analysis of spatial autocorrelation among mtDNA haplotypes. At 50 m – the smallest distance class chosen – the probability that the value of *r* estimated from randomly permuted data would be greater than or equal to the value calculated from the data = 0.0003, and the 95% confidence interval around the estimate of *r* was outside the 95% confidence interval around the null hypothesis of “no spatial structure.” Results at 500 m and 3000 were not significant (*P* at 500 m = 0.956, *P* at 3000 m = 0.636). Thus, colonies separated by 50 m or less were significantly more likely to share the same haplotype than colonies separated by greater distances.

### AFLP data

A total of 205 loci met all our criteria for reliability and diversity. These loci were scored for a total of 399 spiders, including 68 females and 42 males from 13 colonies at Spioenkop Reserve, 49 females and 19 males from 11 colonies at Spioenkop Lodge, 145 females and 60 males from 26 colonies at Weenen, plus one male and 15 females from propagule webs.

### Population structure

We partitioned genetic variation by colony and by collection site with AMOVA, using all colonies for which two or more individuals were fingerprinted (*n* = 50 colonies). Seventy‐one percent of observed variance was accounted for by variation among individuals within colonies, 21% by differentiation among the colonies, and 8% among the three collection sites (Φ_pt_ 0.228, Φ_pr_ = 0.229, Φ_rt_ = 0.076, respectively, *P* = 0.001 for all). Separate analyses for females and males suggested some differences between sexes, with males exhibiting slightly more variation within colonies than females and less among colonies. For females, variation among individuals within colonies accounted for 61% of variance; among colonies, 28%; and among collection sites, 11%. For males, variation among individuals within colonies accounted for 70% of variance; among colonies, 25%; and among collection sites, 5% (Sample sizes and Φstatistics for females: 49 colonies, 261 individuals, Φ_pt_ = 0.395, Φ_pr_ = 0.319, Φ_rt_ = 0.112, *P* = 0.001 for all. For males: 24 colonies, 113 individuals, Φ_rt_ = 0.051, Φ_pr_ = 0.267, Φ_pt_ 0.305, *P* = 0.001 for all.).

AMOVAs retaining colonies as populations but using sex as region suggested differences in population structure of males and females at the Weenen site. We analyzed 56 males in 11 colonies (including only colonies with at least two males) and 144 females in 25 colonies (including only colonies with at least two females) and found 66% of observed variance was accounted for by variation within colonies, 32% by differentiation among colonies, and 3% between males and females (Φ_rt_ = 0.027, *P* = 0.002; Φ_pr_ = 0.324, *P* = 0.001; Φ_pt_ 0.343, *P* = 0.001). However, no significant differences between male and female population structure were detected at the Spioenkop sites.

### Spatial autocorrelation

**Figure 3:**
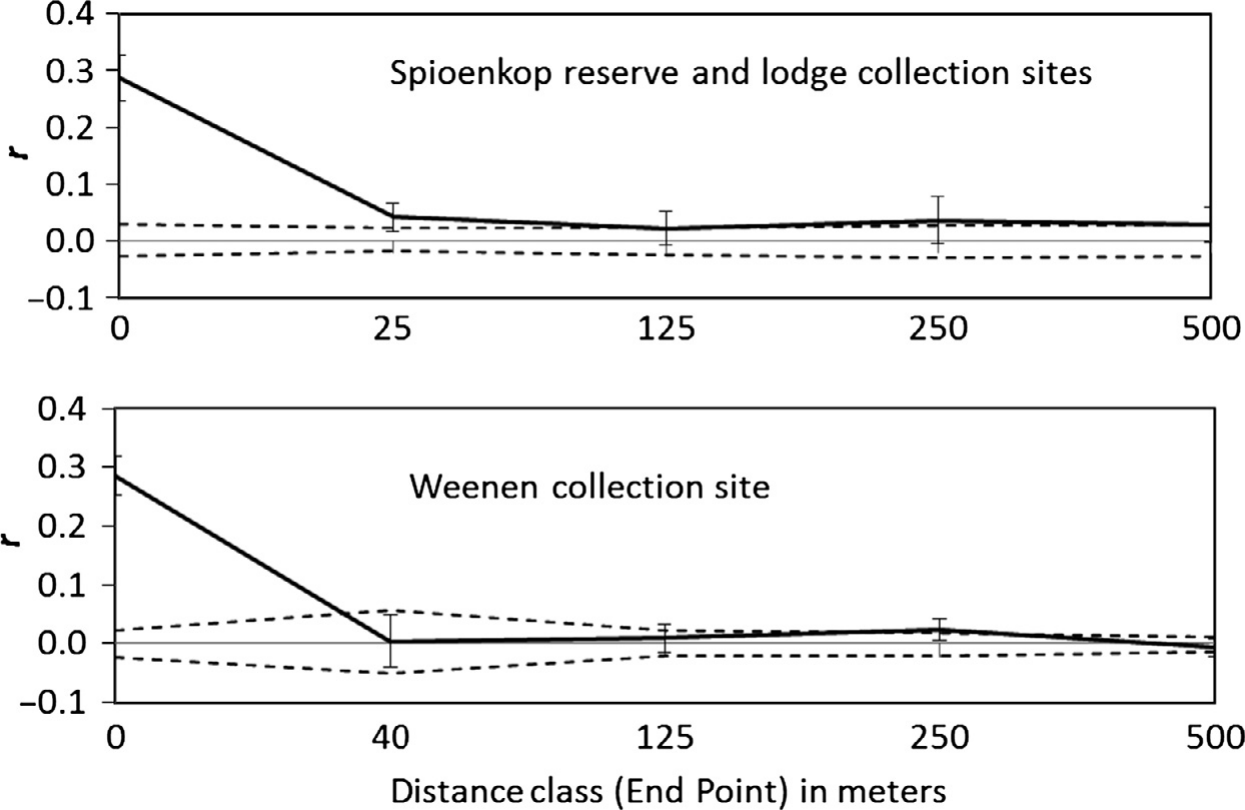
Spatial structure analysis of *Stegodyphus dumicola* (males and females combined) at two South African study sites: above – Spioenkop Reserve and Lodge (62 males, 117 females); below – Weenen (60 males, 145 females). Note the different x‐axes on upper and lower graphs; this was done to accommodate the actual spatial distribution of colonies at the two sites. Solid line = *r*, the autocorrelation coefficient among phenotypes in a given distance class. Dashed lines indicate the 95% confidence interval around the null hypothesis of “no spatial structure” for the data set as determined by 9999 permutations of the data. Error bars indicate the 95% confidence interval about each *r* value, determined by 10,000 bootstrap resamplings. Omega values for combined correlograms indicate significant population structuring at both sites: Spioenkop, Omega = 54.67 (*P* = 0.0002); Weenen, Omega = 36.67 (*P* = 0.001); *P* = the probability Omega values calculated from randomly permuted data are greater than or equal to the observed data.

The combined spatial autocorrelations for males and females at both Weenen and Spioenkop (Fig. 3) showed highly significant deviations from the null hypothesis of no correlation between phenotypic similarity and pairwise distance. In both locations, values of *r* are highest within colonies (distance class 0 m) and decrease with distance.

**Figure 4:**
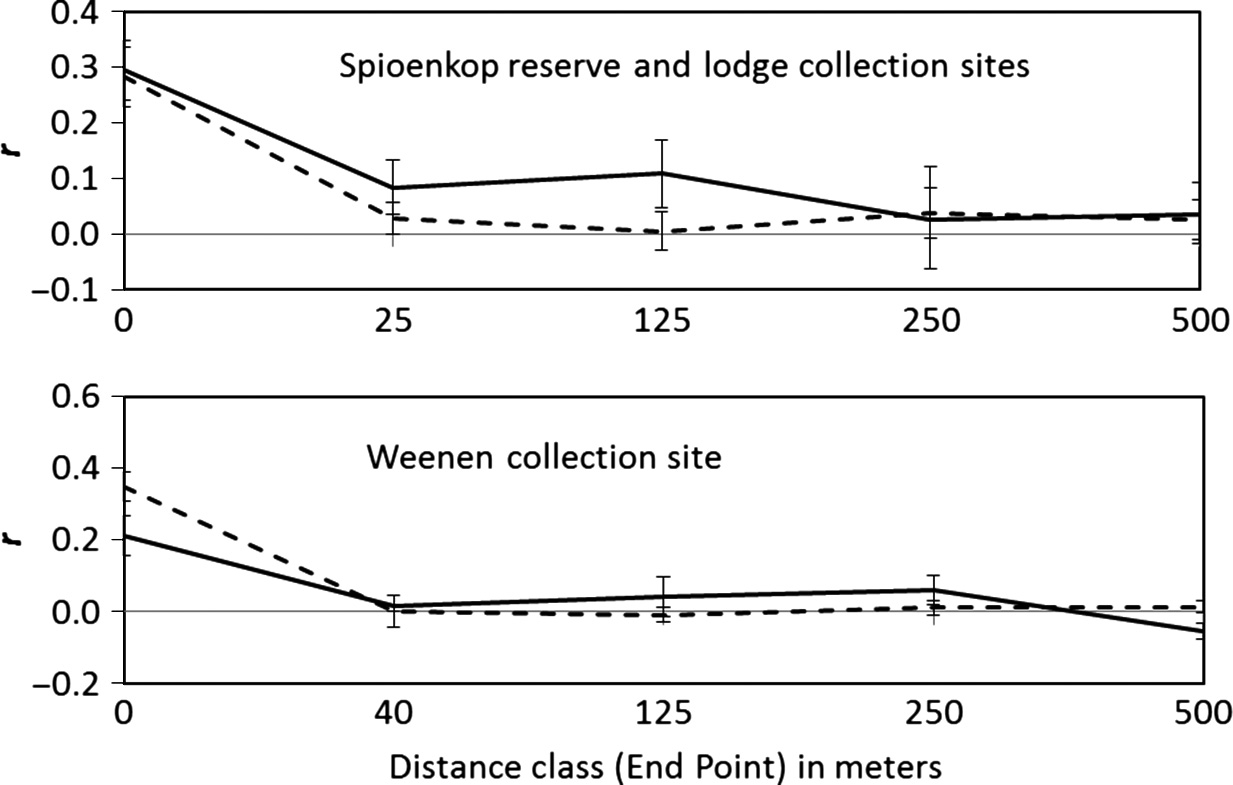
Spatial structure analysis for *Stegodyphus dumicola* males and females analyzed separately: above, Spioenkop Reserve and Lodge (62 males, 117 females); below, Weenen (60 males, 145 females). Males – solid line, females – dashed line, *r** ***= autocorrelation coefficient among phenotypes of males or females in a given distance class; error bars indicate the 95% confidence intervals around values of *r* as determined by 10,000 bootstrap resamplings. Note the different x‐axes on the two graphs. Values of *r* at the Weenen site are significantly lower for males (*r** ***=*** ***0.212) than for females (*r** ***=*** ***0.348) collected from the same colony (T2 at 0 distance class = 12.5, *P* = 0.001). There was no significant difference between male and female colony mates at Spioenkop collection sites (*r* at 0 m distance class = 0.295 for males, 0.284 for females; T2 at 0 m distance class = 0.075, *P* = 0.786)

When tested separately using the Heterogeneity option in GenAlEx, males and females each show significant spatial structure with high values of *r* among colony mates (Fig. 4). At the Weenen site, values of *r* were significantly lower for males than for females collected from the same colony. There was no significant difference in *r* between male and female colony mates at combined Spioenkop collection sites.

### Structure

**Figure 5:**
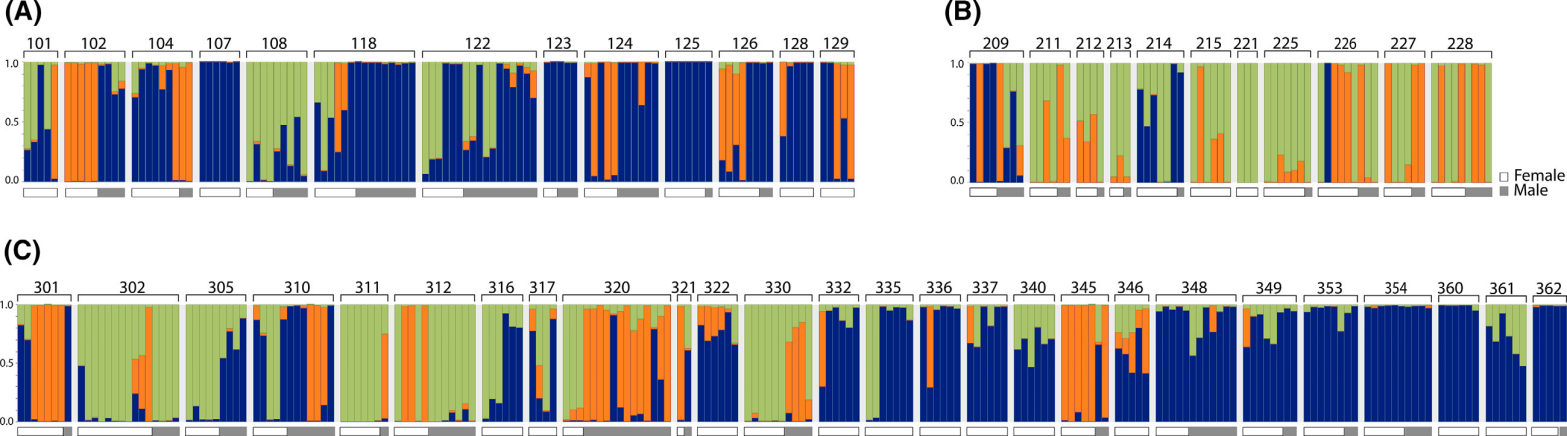
Results of STRUCTURE analysis of AFLP data for three South African populations of *Stegodyphus dumicola*: Spioenkop Dam Nature Reserve (panel A, 100‐series) and Spioenkop Lodge (B, 200‐series) and Weenen (C, 300‐series). Each vertical bar represents one spider, number and parentheses above bars indicate colony number (see Figs. 1 and 2), and horizontal bar below colony mates indicates females (black) and males (gray). For all three populations, the most probable number of ancestral sources contributing to the sampled population is *K* = 3. Colors for each individual indicate probability of descent from one of three ancestral sources. Each collection site was analyzed individually, so the three ancestral sources cannot be assumed to be the same across all three collection sites.

At all three collection sites – Spioenkop Reserve, Spioenkop Lodge, and Weenen – *K* = 3 were selected as the most probable number of “ancestral” or initial source populations. Figure 5 illustrates that the assignments of ancestry differ among individuals both within and among colonies in a collection site, complementing the findings of AMOVA.

Using the model “ancestry proportions = constant + sex + colony,” we examined whether an individual's probable ancestry differed significantly among colonies, or between males and females. This model was significant for samples from Spioenkop Reserve and Weenen, but not for samples from Spioenkop Lodge (Table 1). At the Spioenkop Reserve and Weenen sites, colony identity significantly influenced an individual's probability of descent from each of the three putative source populations (Table 2). At Spioenkop Reserve, sex also had a significant influence on an individual's assignment to each of the source populations.

**Table 1:** Maximum‐likelihood fit of probable ancestry of *Stegodyphus dumicola* (see Results of STRUCTURE analysis in text and Fig. 5) to the model “ancestry proportions = constant + sex + colony,” where *K*, the most probable number of ancestral populations contributing to the sampled population, is 3

Location	Spioenkop reserve	Spioenkop lodge	Weenen
Sample size	111	68	205
ML fit of fractional multinomial logit: log pseudo likelihood	−91.934819	−59.003542	−176.74498
Wald *χ* ^2^	21.42	6.98	101.35
Probability of a larger value of *χ* ^2^	0.003	0.1368 ns	0

**Table 2:** Fit of the model, “ancestry proportions = constant + sex + colony” to STRUCTURE predictions for the Spioenkop Reserve and Weenen collection sites; the Spioenkop Lodge collection site is not included here as the model was not significant for that data set (see Table 1)

Collection site	Putative source	Model component	Coefficient	Robust std. error	*z*	*P* > |*z*|	95% confidence interval	
Spioenkop Reserve	Source 2	Sex	−1.522	0.538	−2.83	**0.005** a	−2.577	−0.467
		Colony	−0.034	0.026	−1.29	0.197	−0.086	0.018
		Constant	4.694	2.971	1.58	0.114	−1.129	10.517
	Source 3	Sex	−0.136	0.438	−0.31	0.756	−0.994	0.721
		Colony	−0.063	0.019	−3.35	**0.001** a	−0.100	−0.026
		Constant	6.068	2.203	2.75	0.006	1.550	10.385
Weenen	Source 2	Sex	0.532	0.366	1.46	0.145	−0.184	1.245
		Colony	−0.053	0.010	−5.48	**0.000** a	−0.072	−0.034
		Cons	15.725	3.210	4.90	0.000	9.434	22.016
	Source 3	Sex	−0.401	0.320	−1.26	0.209	−1.028	0.225
		Colony	−0.071	0.008	−9.34	**0.000** a	−0.086	−0.056
		Constant	23.247	2.557	9.09	0.000	18.234	28.259

a Bold‐faced values indicate significant effect of sex or colony membership on ancestry proportions.

## Discussion

We used both maternally inherited mtDNA and biparentally inherited, dominant nuclear markers (AFLP fingerprints) to investigate sex‐specific dispersal in the cooperative social spider *S. dumicola*. By analyzing adult females and males collected during and after the mating season, we were able to detect whether mating dispersal is undertaken by either sex.

In agreement with earlier work, we found that colonies comprise individuals sharing a common mtDNA haplotype (Johannesen et al. 2002, 2007), and variation in the nuclear genome is partitioned primarily among colonies (Smith et al. 2009). The broad picture is of limited movement among established colonies by individuals of either sex. Over time, this would be expected to cause loss of genetic variation within colonies and colony lineages through drift, and divergence among colony lineages. This could be counteracted by gene flow among nests through movements of either males or females.

Earlier experimental studies demonstrated that adult males could travel among nests over distances up to 7 m (Lubin et al. 2009). Here, we detected significant genetic evidence of male dispersal among colonies, which would act to counter effects of drift within nests. We also detected evidence of medium‐distance (within collection site) and long‐distance female dispersal, which contributes to maintenance of genetic similarity among populations.

The observed mtDNA haplotype distributions in the Weenen population (Fig. 2) provide evidence of both long‐ and medium‐range dispersal by females. Long‐distance dispersal is indicated by the presence of haplotypes from two mtDNA lineages, indicating that the population was initiated by at least two females with different mtDNA haplotypes (with possible *in situ* mutations to create four haplotypes). Medium‐distance female dispersal is indicated by the distribution of mtDNA haplotypes at the Weenen site, and by the mtDNA haplotypes of females in propagule webs. We found that the closest pairs of colonies (separated by 50 m or less) had the same mtDNA haplotypes, consistent with colony proliferation by budding. At nearest neighbor distances greater than 50 m between colonies, we observed a mosaic of colony haplotypes, consistent with medium‐range dispersal of females by ballooning, and (in older populations) extinction of colonies.

While most of the spiders in propagule nests were females carrying the same mtDNA haplotype as the nearest large colony, in two cases the dispersing females carried a mitochondrial haplotype different from that of the nearest large colony, indicating they originated in a more distant colony. Nonetheless, in the Weenen population all colony mates examined, whether male or female, shared the same mtDNA haplotypes, even if colonies with different haplotypes were nearby. This suggests that ballooning females do not routinely join established colonies and that dispersal by reproductive males may take place primarily among nearby colonies, which are likely to share the same mtDNA haplotype.

One male was found in a propagule nest, showing natural male dispersal (see also Lubin et al. 2009). As propagule nests consist of recently dispersed females, the male was not an offspring, but rather clearly a disperser. If adult males sometimes leave their natal colonies and disperse to other colonies in search of mates, then males in a colony will – on average – be less similar to one another than females from the same colony. In addition, the decline in autocorrelation values over distance should be steeper for females than for males. Using TE‐AFLP data, we found significantly lower values of *r*, the coefficient of similarity, among male colony mates than female colony mates at the Weenen site, though not at the Spioenkop sites. At both Weenen and Spioenkop, the decline in *r* is steeper for females than for males (Fig. 4), also consistent with male among‐colony mating dispersal. These results suggest that male short‐distance dispersal among colonies may play a greater role than female propagule dispersal in maintaining genetic variation within *S*. *dumicola* colonies.

STRUCTURE analysis provides a visual demonstration that colony mates are not genetically uniform (Fig. 5), where individuals could be assigned to up to three possible ancestral sources in each of the three collection sites. Colonies differed in the number of ancestral sources (1, 2, or all 3) represented among colony mates, and the proportion of individuals of inferred mixed ancestry, that is, with a probable assignment to more than one ancestral source. At Spioenkop Reserve, there was a significant difference in ancestries inferred for males and females, again consistent with movement of males. We propose that males move from their natal colony, perhaps after all females in their home colony have been inseminated, and seek to mate with any available females in an adjacent colony. The process is repeated in subsequent generations, resulting in a small amount of gene flow through males within the local population. As males are unlikely to move larger distances (they do not balloon), the likelihood of extreme outbreeding is low. In fact, long‐distance outbreeding could even be deleterious if it disrupts combinations of alleles that produce a locally well‐adapted phenotype. Indeed, an experimental breeding study (Berger‐Tal et al. 2014) showed negative fitness effects of breeding between males and females collected from different populations.

Gene flow and dispersal among colony lineages of *Stegodyphus dumicola* are low compared with outbreeding populations, but nonetheless dispersal by both males and females does take place. Short‐distance proliferation by colony budding, and short‐ and medium‐range female dispersal by ballooning are responsible for initiating new nests in local populations; long‐distance dispersal by ballooning is responsible for initiation of new populations. While direct observations were rare, genetic analyses confirmed male dispersal. Adult male dispersal appears to be primarily over short distances and may be important for gene flow among nearby colonies, thereby maintaining genetic cohesion among colonies in local populations. Thus, of the four proposed means of gene flow, we have been able to confirm only two, namely joining up of dispersing propagule females (observed in the field) and short‐distance moves of males (observed, and shown here with genetic data). We found no evidence for either colony fusion or introgression by ballooning females into established colonies. Rather, the haplotype data suggest that these behaviors must be rare, at least when colonies are widely spaced.

Social spider population structure shows a level of inbreeding seen in few other nonclonal organisms. Naked mole rats, dwarf mongoose and ambrosia beetles, have regular inbreeding, but also at least occasional outbreeding (Reeve et al. 1990; Keane et al. 1996; Peer and Taborsky 2005). Given a choice, naked mole rats and seed beetles prefer to outbreed rather than to mate with kin (Clarke and Faulkes 1999; Gottlieb et al. 2009). No such preference is seen in social spiders, nor is there any evidence of inbreeding depression (Berger‐Tal et al. 2014). Yet a non‐negligible amount of within‐colony genetic variation exists (Smith et al. 2009; this study), and a low level of gene flow through local, male‐biased dispersal may explain how this variation is maintained. The question remains as to the importance of such variation in the maintenance of the inbred social system.

## Conflict of interest

The authors declare that they have no conflict of interests related to this study. This is publication no. 906 of the Mitrani Department of Desert Ecology.
